# The Faith Care Family Project: A Pilot Intervention for African American Dementia Family Caregivers

**DOI:** 10.1177/15333175251363706

**Published:** 2025-08-03

**Authors:** Noelle L. Fields, Ling Xu, Ishan C. Williams, Fayron Epps, Samantha Tinker

**Affiliations:** 1 School of Social Work, 12329 The University of Texas at Arlington, Arlington, TX, USA; 2 School of Nursing, 2358 University of Virginia, Charlottesville, VA, USA; 3 UT Health San Antonio, San Antonio, TX, USA

**Keywords:** family caregiving, volunteer, psychoeducation, African American

## Abstract

The Faith Care Family (FCF) Project was a telephone based, volunteer-led intervention for African American Alzheimer’s disease and related dementias (AD/ADRD) family caregivers that was piloted in one predominantly African American church. Focus groups with faith leaders and parishioners informed the training and intervention. Eighteen church volunteers participated in the FCF Project training, but 9 of them were paired with family caregivers and completed the FCF Project intervention. Church volunteers indicated overall significant improvements knowledge of AD/ADRD after the training as well as after the intervention. Quantitative results indicated that caregivers increased their knowledge of dementia, reported improved coping skills, and reported increased positive aspects of caregiving. Feeling a connection, normalizing the challenges of caregiving, gaining or reinforcing knowledge, and sharing community resources were themes from qualitative interviews with the family caregivers. Overall, findings indicate that the FCF Project shows promise as an intervention for African American AD/ADRD family caregivers.

## Introduction

Alzheimer’s disease and related dementias (AD/ADRD) rank among the most complex chronic health issues in the United States. The number of Americans aged 65 and older affected by AD/ADRD is expected to increase from 58 million in 2021 to 88 million by 2050.^1^ There are significant disparities in ADRD diagnosis and prevalence between older African American populations and white Americans.^2^ These disparities also affect family caregivers who provide most of the care for persons living with ADRD in non-institutional settings.^2^ Family caregivers not only assist with daily activities but also face managing the challenging symptoms that often accompany the cognitive and behavioral changes in persons living with AD/ADRD. Research further suggests that racism and discrimination, cultural expectations, financial challenges, and concerns about environmental safety create a distinct and challenging experience for African American AD/ADRD caregivers.^2-4^

Although numerous psychosocial and psychoeducational interventions have been empirically validated for AD/ADRD family caregivers,^5-7^ there remains a need for culturally-relevant, community-based interventions for African American AD/ADRD caregivers.^4^ To better support African American AD/ADRD family caregivers, research suggests that interventions should prioritize cultural relevance and cultural tailoring.^8,9^ For example, in some African American families, caregiving may be viewed as a normative and expected role rather than a life disruption.^4^ Thus, researchers recommend that studies should be cautious using the term caregiver ‘burden’ as it may not fully capture the cultural experience of caregiving.^4,10^ Other research underscores that some African American caregivers focus on the enduring aspects of the AD/ADRD care recipient’s personality, rather than primarily mourning the losses associated with AD/ADRD. This focus may be shaped by ancestral family values and cultural traditions grounded in resilience and shaped by historical experiences of oppression.^11^

Growing evidence supports the use of lay provider interventions, particularly those designed collaboratively and culturally tailored to the specific needs of local communities, as this approach may better engage and address the needs of African American AD/ADRD dementia caregivers.^12-15^ In a study aimed at increasing AD/ADRD awareness, detection, and diagnosis among rural, racially and ethnic diverse older adults, the involvement of local faith health educators to serve as lay health advisors was reported as central to the intervention’s success. Notably, the community-based initiative continued to be sustained independently of the research team, highlighting the potential long-term impact of lay providers.^12^ The lay provider model also increases accessibility and strengthens translational potential, as most evidence based AD/ADRD caregiver interventions require interventionists to possess professional degrees or academic credentials.^16^

There is a continued need to improve the accessibility of AD/ADRD family caregiver interventions. A common limitation of many studies is that AD/ADRD family caregivers often struggle to participate in face-to-face interventions due to their inability to leave the care recipient unattended, physical constraints, or residing in areas with inadequate infrastructure.^17,18^ Telephone-based interventions may enable AD/ADRD family caregivers to take part in sessions more flexibly as part of their daily activities, which may help to overcome barriers to access and participation.^17,18^ Given the lack of AD/ADRD specialists in rural areas, telephone based approaches may also offer a more accessible option for family caregivers living in these communities.^19^ A meta-analysis indicated that telehealth interventions provided significant benefits for individuals living with dementia (reducing depressive symptoms) and their family caregivers (enhancing perceived competence).^20^ Similarly, a systematic review focused on using telehealth interventions for AD/ADRD family caregiver found significant benefits in reducing caregiver burden, depression, and stress.^21^ Nevertheless, studies in these reviews involved the use of trained interventionists rather than lay providers, underscoring an opportunity for further research.

In particular, faith-placed settings such as churches may offer a promising platform for the design and delivery of lay provider, interventions for African Americans,^22^ including programs designed for ADRD family caregivers.^23,24^ African American family caregivers often rely on religion/spirituality, prayer, and faith in God as a specific resource for coping.^25^ There is also evidence that attending church may enhance the wellbeing of African American AD/ADRD caregivers^26^ and religious participation may play a role in ameliorating AD/ADRD risk for some African Americans adults.^27,28^ Research exploring the church as a platform for raising AD/ADRD awareness suggests that churches are well-equipped to offer support and services to families impacted by AD/ADRD.^29^

Expanding on the promise of faith-placed platforms for interventions, faith-integrated interventions offer a culturally responsive approach to supporting Black/African American AD/ADRD caregivers by leveraging the central role of spirituality and faith-based institutions (eg, churches) in Black/African American communities.^30^ Black/African American families often turn to their faith communities as trusted sources of social and emotional support.^31,32^ Integrating faith-based elements into caregiver support programs can increase cultural relevance, challenge stereotypes about mental health, and enhance trust and engagement with services for AD/ADRD that might otherwise be met with skepticism.^15,24,33^ Faith-based elements not only improve retention in caregiving interventions but may also help to address longstanding barriers to care including medical mistrust stemming from historical and ongoing systemic inequities.^34^

Research suggests a growing need for virtual health ministry models that include telehealth in African American religious communities.^35^ Two notable studies that have used a telephone intervention to support African American AD/ADRD caregivers include Tele-Savvy^36^ and ACTS2.^14^ Tele-Saavy involved 7 synchronous Zoom sessions with small groups of AD/ADRD caregivers led by 1 or 2 trained (ie, professional) facilitators. While the program led to significant improvements in caregiver mastery and depression for white participants, these benefits were not observed among Black/African American participants. ACTS2 was a faith-integrated intervention for African American AD/ADRD caregivers consisting of 7, 1-hour small group sessions and 5, 1-hour individual sessions. Sessions were led by trained faith community workers, called lay pastoral care facilitators, who volunteered to provide direct care and peer-support services to their local congregations and communities. Findings from ACTS2 indicated that caregivers experienced significant improvements in depression, health status, problem severity, and social support.^14^ Qualitative findings underscored the value of relationships with co-participants and facilitators, the central role of spirituality, and importance of goal setting.^14^

Building on these insights and on studies of another lay provider, African American AD/ADRD caregiver intervention, the current study aimed to adapt and pilot test a telephone-based, volunteer-led, psychoeducational intervention for African American AD/ADRD family caregivers in a faith-placed setting. Framed by the revised sociocultural stress and coping model which highlights the interaction of cultural values with key elements of the stress and coping model,^37^ the proposed Faith Care Family (FCF) Project integrates an adapted lay provider intervention for AD/ADRD family caregivers in a predominantly African American church to address caregiver burden, coping skills, and social support.

The Faith Care Family (FCF) Project contributes new knowledge to the field of dementia caregiving interventions by introducing and testing a novel, telephone-based, volunteer-led, and one-on-one psychoeducational model that is uniquely adapted for African American AD/ADRD family caregivers within a faith-placed setting. While previous studies like Tele-Savvy and ACTS2 have demonstrated the potential of group-based and faith-integrated approaches, the FCF Project diverges in 4 ways that contribute new insight for AD/ADRD interventions. First, it emphasizes individualized, dyadic support rather than group sessions, potentially offering a more personalized model. Second, unlike ACTS2’s reliance on trained lay pastoral care facilitators or Tele-Savvy’s use of professionals, the FCF Project equips untrained church volunteers to serve as lay providers, highlighting the feasibility of engaging community members without prior caregiving or pastoral experience. Third, the FCF Project is faith-placed rather than faith-based, meaning it situates caregiving support within the church community context while maintaining a secular core curriculum. This approach respects the cultural and spiritual significance of the church for African American families without explicit inclusion of religious content (eg, prayer, scripture readings, faith sharing), potentially broadening its acceptability and application in the larger community. Fourth, by grounding the intervention in the revised sociocultural stress and coping model, the FCF Project adds theoretical depth to how cultural values interact with caregiver stress processes. Through its three-phase design focusing on community-informed adaptation, evaluation of training and intervention outcomes, and qualitative feedback, the study also demonstrates a replicable, community-engaged framework for AD/ADRD caregiver interventions.

The FCF Project was implemented in 3 phases and sought to answer the following research questions:

(Phase 1: inform the adaptation of the intervention)

*How do faith leaders and parishioners perceive the key components of the FCF Project and what are their recommendations for implementation?*

(Phase 2: test the preliminary efficacy of the training)

*Do the church volunteers (*eg*, lay providers) gain knowledge of AD/ADRD after the 1-day, FCF Project training?*

(Phase 2: test the preliminary efficacy of the intervention)

*Do African American AD/ADRD family caregivers demonstrate more knowledge of dementia, less caregiver stress and improved well-being, more social support, and better coping skills after they participate in a lay provider intervention?*

(Phase 3: enrich the interpretation of the results of the intervention)

*How do participants experience the FCF Project?*

## Methods

This study used a multi-phase, mixed methods design.^38^ In Phase 1, qualitative data were gathered through focus groups to inform the development of the FCF Project and training in Phase 2. In Phase 3, individual interviews were conducted to enhance the interpretation of the intervention outcomes from Phase 2.

The research team designed a three-phase, mixed methods approach based on the strong evidence suggesting that integrating qualitative and quantitative methods is particularly well-suited to the development of socio-culturally grounded interventions.^39^ Mixed methods allow for the collection of both broad patterns and rich, contextualized data, which is especially valuable when working with populations disproportionately affected by Alzheimer’s disease and related dementias (AD/ADRD).^40^ The sequential structure of this design, where each phase informs the next, creates a feedback loop that enables iterative refinement, enhances cultural and contextual relevance, and supports the real-world applicability of the intervention. Additionally, this methodology builds upon the research team’s prior work using a similar three-phase design to adapt a lay provider intervention for African American AD/ADRD caregivers which was successfully implemented in a secular community-based setting.^13^

**Figure 1.**
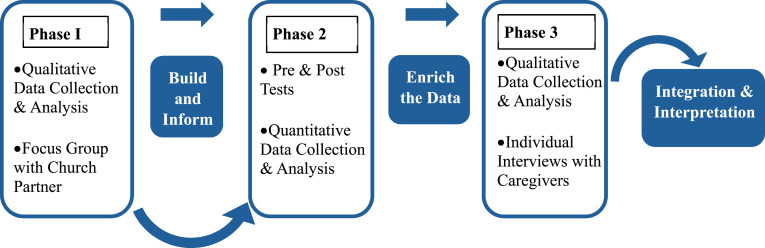
Multi-phase, Mixed Methods Design of the FCF Project (Adapted From Creswell & Plano Clark, 2018)

The mixed methods design also addressed the 2 components of the updated sociocultural stress model^37^: the common core, assessed through the coping scales, and the culturally specific domains, explored through interviews with participants. A member of the partner church who possessed knowledge of volunteer-led interventions for AD/ADRD caregivers and had a strong, trusting social network in the local African American community^41^ functioned as a community liaison and member of the research team. The 3 phases of the study are described in Figure 1.

### Phase 1

The intent of Phase 1 was to gather feedback and insight to build and inform the intervention implementation in Phase 2. Two focus groups were conducted with faith leaders and parishioners at one predominantly African American church located in a large metropolitan city in North Texas. The church was selected because of the research team’s community liaison, who was a member of the church and connected the research team to the pastor. The community liaison had also previously collaborated with the research team on other community-based studies related to AD/ADRD.^13^ The overall goal of Phase 1 was to gather information from the church partner to adapt a lay provider psychoeducational intervention for African American AD/ADRD caregivers that previously utilized volunteers from the Senior Companion Program.^13,42,43^

### Phase 2

Phase 2 involved a FCF Project training and FCF Project intervention. Purposive sampling was used to recruit Black/African American church volunteers and Black/African American AD/ADRD family caregivers located in a large metropolitan city in Texas. The church leaders from Phase 1 assisted with sharing a study flyer with parishioners as well as with their local community contacts (eg, other churches, social service agencies) and provided information about the study after worship services.

The research team included a community liaison, who was a member of the church from Phase 1 and who had previously collaborated with the research team on other community-based studies related to AD/ADRD, also assisted with recruitment. To be eligible for this study, church volunteers were: (1) affiliated with the church from Phase 1; (2) self-identified as Black/African American; and (3) were at least 18 years of age. The caregivers were: (1) self-identified as Black/African American; (2) at least 18 years of age; and (3) currently providing informal care for a community-dwelling person with ADRD.

### Phase 3

Phase 3 interviews, conducted in June and July 2023, aimed to enhance the interpretation of the Phase 2 results. The individual qualitative interviews with the family caregivers from Phase 2 lasted approximately 30-60 minutes. A semi-structured interview guide, consisting of open-ended questions, was used to explore participants’ experiences with the FCF Project following the final intervention module. Questions included: (1) What did you learn about dementia and Alzheimer’s disease? (2) How is your level of stress as a caregiver different? (3) Do you cope with your role as a caregiver differently? If so, how?; and (4) What are some ways that you take care of your family member with dementia differently?

## Data Collection

### Phase 1

Prior to data collection, focus group participants (*N* = 20) completed an informed consent form on QuestionPro that was approved by the Institutional Review Board of the University. The research team engaged faith leaders (*n* = 6) and parishioners (*n* = 14) at one predominately African American church. To develop a culturally informed and logistically sound psychoeducational intervention for AD/ADRD family caregivers, the church was a collaborative partner in the design of the FCF Project. The research team gathered feedback and information through 2, 90-minute focus groups on Microsoft Teams in December 2022 and January 2023. For consistency, the same 3 researchers facilitated both groups while 2 other researchers took notes. Using a semi-structured focus group guide, participants were broadly asked questions based on a volunteer-led AD/ADRD intervention from which the FCF Project was adapted^13,42,43^: (a) What components of the FCF Project are important to you? and (b) How can the FCF Project be modified for your church? See Table 1 for sample characteristics of the focus group participants.

**Table 1.** Demographics of Focus Group Participants

Variables	Focus group 1_Leaders (n = 6)		Focus group 2_Affiliates (n = 14)	
	Median [Range]	n (%)	Median [Range]	%
Age	62.00 [47-75]		51.5 [25-79]	
Sex_female		5 (83.3%)		13 (92.9%)
Education				
Some college or below		2 (33.3%)		9 (64.3%)
Bachelor’s degree		1 (16.7%)		5 (35.7%)
Graduate or professional degree		3 (50.0%)		
Length in church (years)	20.50 [0.25-50]		32 [0.1-69]	
Frequency of attending church				
Once a week		2 (33.3%)		12 (85.7%)
More than twice a week		4 (66.7%)		2 (14.3%)
Have personal experience with AD/ADRD (yes)		1 (16.7%)		8 (57.1%)
Be aware of any church members having AD/ADRD (yes)		3 (50.0%)		1 (7.1%)
Be aware of any church members who are AD/ADRD caregivers (yes)		5 (83.3%)		1 (7.1%)
Church provided AD/ADRD related education/programs/services (yes)		0 (0%)		0 (0%)
Church provided education/programs/services for AD/ADRD caregivers (yes)		0 (0%)		0 (0%)

### Phase 2

Prior to data collection, church volunteers completed an informed consent form on QuestionPro and family caregivers gave verbal consent by phone using a form approved by the Institutional Review Board of the University. Although 18 church volunteers participated in the training, only 9 were paired with family caregivers due to a shortage of recruited caregivers. In this paper, we presented the findings from the 9 dyads who completed the intervention phase of the study. Table 2 provides the sample characteristics of the church volunteers.

**Table 2.** Church Volunteer Participants Demographics (N = 9)

	n	Median [Range]	%
Age	9	66 [26-92]	
Sex_female	8		100%
Degree in progress			
Some college or below	4		33.30%
Associate/Bachelor’s/Master’s degree	5		66.60%
Marital status			
Never married	3		33.30%
Married/cohabiting	2		22.20%
Widowed	2		22.20%
Divorced	2		22.20%
Financial strain			
Not difficult	7		77.80%
Difficult	2		22.20%
Self-rated health			
Fair	2		22.20%
Good	4		44.40%
Very good or excellent	3		33.30%
Religiosity	9	25.0 [19-27]	

Phase 2 began with a 1-day, 7-hour, in-person training for church volunteers in February 2023. The FCF Project training was conducted as an in-person, group workshop, incorporating both didactic and interactive activities (eg, role play) across 6 topics (See Table 3 for an overview of the FCF training). Each church volunteer received a printed FCF Project Manual. Breakfast and lunch were provided as part of the training. Three members of the research team facilitated the training, and 2 graduate research assistants were present to take notes. Pre- and post- questionnaires were administered to assess whether participation in the FCF Project training enhanced the church volunteers’ knowledge of AD/ADRD.

**Table 3.** The Content of the FCF Training

Module	Topic
1	Myths and facts about Alzheimer’s disease
	1) Overcome stigma 2) Separate the facts from the myths about the disease 3) Provide facts about types and symptoms of AD 4) Support the independence and promote the dignity of persons with AD
2	Home safety, personal care, and behavior
	1) Create a safe environment for a person with AD, including ways to prevent falls 2) Provide information about ways to assist in the maintenance of daily personal care (bathing and toileting, oral hygiene, dressing, grooming, eating, incontinence) 3) Develop strategies to avoid problematic behaviors (wandering or trying to leave, resistance to care, combativeness, sleep disturbances, threatening or calling out, and hoarding)
3	Health care visit and community-based services
	1) Provide ways to overcome barriers to communication with health care professionals 2) Prepare for the health care visit 3) Improve communication with health care professionals 4) Provide strategies for organizing information during health care visit 5) Identify options for those in need of assistance within the community 6) Provide information on how to access services
4	Communication skills with persons with family members
	1) Learn about the communication changes that occur in a person with AD 2) Develop practical skills to enhance ongoing communication with a person with AD at different stages 3) Discuss strategies for communicating with other family members, friends, and neighbors about dementia
5	Coping skills for the emotional consequences associated with caregiving
	1) Recognize and understand the range of emotions that accompany caregiving 2) Discuss strategies to cope with negative emotions 3) Develop strategies for expressing emotions and mental health 4) Adjust emotional expectations as a caregiver
6	Coping with expectation and finding meaning and purpose in caregiving
	1) Discuss changes that may occur in family relationships as a result of caring AD 2) Understand and resolve family conflicts 3) Find cultural values/satisfaction in caregiving 4) Examine aspects of spirituality and caregiving 5) Reflect on the positive aspects of caregiving 6) Explore aspects of caregiver resiliency
7	Summary of modules 1-6

Following this training, the church volunteers were randomly matched with one AD/ADRD family caregiver to begin the 6- week, telephone-based intervention (ie, 1:1 match). Caregivers were mailed a copy of the FCF Project manual prior to the start of the intervention. Volunteers and caregivers were given each other’s phone numbers. The dyads were asked to meet once a week by telephone (approximately one hour), for 6 weeks. During each weekly call, the dyad discussed one module using the FCF Project Manual (refer to Training Modules 1-6 in Table 3). The manual included “ice breaker” questions at the beginning of each module to build trust and rapport.

As part of Phase 2, 9 AD/ADRD family caregivers participated in the FCF Project telephone-based intervention (See Table 4 for caregiver demographics) in April and May 2023. Pre-post questionnaires were used to examine if the AD/ADRD family caregivers who participated in the 6-week intervention improved their knowledge of dementia, caregiver burden and/or stress, coping skills, social support, and well-being. Data were collected by phone and entered into QuestionPro by a member of the research team at baseline prior to the intervention, at the end of the intervention, and 3- months post-intervention.

**Table 4.** Caregiver Participants Demographics (N = 9)

	n	Median [Range]	%
Age	9	62 [42-52]	
Sex_female	8		100%
Education			
Some college or below	5		66.60%
Bachelor’s/master’s degree	4		33.30%
Marital status			
Never married	2		22.20%
Married/cohabiting	4		44.40%
Widowed/divorced/others	3		33.30%
Live with CR (yes)	6		66.70%
Employment (full time)	4		44.40%
Relationship with CR			
Spouse	2		22.20%
Children	2		22.20%
Others	5		55.60%
Caregiving length (years)	9	5 [1.5-10]	
Caregiving intensity (hours)	9	10 [0-24]	
Financial strain			
Not difficult at all	4		44.40%
A little difficult	3		33.30%
Very difficult	2		22.20%
Self-rated health of CR			
Poor	2		22.20%
Fair	2		22.20%
Good or very good	5		55.50%
Self-rated health of CG			
Fair	3		33.30%
Good	3		33.30%
Very good or excellent	3		33.30%
CR dementia severity	9	27 [16-56]	
Religiosity	9	23.0 [12-27]	

### Phase 2 Measures

The primary outcome measure for the church volunteers was knowledge of dementia measured by the Alzheimer’s Disease Knowledge Scale (ADKS).^44^ The scale consists of 30 true/false items covering topics such as life impact, risk factors, symptoms, treatment and management, caregiving, assessment, diagnosis, as well as course.^44,45^ Research has shown the ADKS to be reliable and have validity in evaluating knowledge about AD/ADRD.^44^ Participants received 1 point for each correct response and 0 points for incorrect answers. The individual average correct response rates for the 30 items, as well as the total correct scores, will be calculated.

In addition to knowledge of AD/ADRD using the ADKS, the primary outcome measures for the family caregivers included: caregiver stress and/or burden, coping skills, social support and activity, satisfaction with social support, caregiver appraisal of benefits, cultural justifications for caregiving, and caregiver well-being. Dementia caregiver burden and/or stress was measured by the Zarit Burden Interview (ZBI) with 22 items.^46^ The items are rated on a scale from 0 to 4, with higher scores reflecting greater levels of distress. Total scores range from 0 to 88. The ZBI has demonstrated strong validity and reliability among diverse minority dementia caregivers,^47^ including among African American caregivers.^48^

*Coping skills* were assessed by Brief Cope subscales with 28 items.^49^ This scale was used to measure the ways/strategies caregivers have been coping with the stress in their life with a 4-point scale ranging from “I have not been doing this at all” (1) to “I have been doing this a lot” (4). Studies show good validity and reliability (range from 0.72 to 0.84 across the subscales) using the Brief Cope in caregivers of persons with dementia.^49^

*Social support and activity* were measured by received support and satisfaction with support. Received social support for caregiving was measured by asking caregivers whether they got 7 support items for their caregiving (1 = yes, 0 = no).^50^

*Satisfaction with social support* was measured by asking caregivers 4 questions about how they feel satisfied with the support they received in the past month from friends, family, and others.^51,52^ Each item was measured with 3 Likert scale ranged from (0) ‘Not at all’ to (3) ‘very’. Research has shown this measure to be valid and reliable with ethnic minority dementia caregivers.^53^

*Caregiver appraisal of benefits* was assessed with the Positive Aspects of Caregiving (PAC) scale^54^ with 9 items. The PAC consists of 9 items phrased as statements about the caregiver’s mental-affective state in relation to the caregiving experience. Each item is rated on a 5-point Likert scale ranging from “disagree a lot” (1) to “agree a lot” (5). The PAC is a valid and reliable measure for different ethnic minority dementia caregivers with Cronbach’s alpha of .85,^47^ and especially useful for African American dementia caregivers.^55^

*Cultural justifications for caregiving* were measured by the Cultural Justifications for Caregiving Scale (CJCS),^56^ which is a 10-item measure designed to assess caregivers’ cultural reasons and expectations in providing care. Each item is measured using a 1-4-point Likert scale ranging from “(1) strongly disagree” to “(4) strongly agree.” Summed scores were used which range from 10 to 40, with higher scores indicating stronger cultural reasons for giving care. Results have shown good reliability and validity score of this scale with African American caregivers.^56^

*Caregiver well-being was* measured by the 16 items short-form Caregiver Well-Being Scale (CWBS).^57^ The CWBS(^58^) was developed to help family caregivers, clinicians, and researchers to identify areas of caregiver strength and areas in which additional support is needed. All the 16 items are measured by a 5-point scale ranging from “rarely” (1) to “usually” (5). Studies show that the short version CWBS has good validity and overall reliability coefficient, regardless of the ethnicity of the caregivers.^57^

### Phase 3

Individual interviews were transcribed by Microsoft Teams and subsequently checked by a trained graduate student. To protect the identity of the family caregivers, pseudonyms were assigned to each participant.

## Data Analysis

### Phase 1

After the focus groups were transcribed by Microsoft Teams and verified by for accuracy by a trained graduate student. The student verified the accuracy of the transcriptions by comparing them with the audio recordings to ensure that the context and intended meaning of participants were accurately preserved for rigorous data analysis.^59^ Next the data were analyzed using the six-step thematic analysis outlined by Braun and Clarke.^60^ This approach enabled the researchers to examine broader conceptual topics, such as the FCF Project module content as well as future applications of the program. Initially, the lead author reviewed the interview transcripts and recordings to become familiar with the data (step 1). The lead author then applied inductive, open coding to the data (step 2), generating codes that captured the meanings conveyed by participants in their own words.^61^ The open coding was conducted using the comments function in Microsoft Word which enabled the researcher to highlight relevant text and record corresponding codes in the side margins.^62^ All of the coded data were then reviewed and collated into preliminary themes using a thematic map^60^ (step 3). These initial themes were independently reviewed by the second author (step 4). Subsequently, the 2 researchers met to refine the data into 4 themes, aligned them with the goals of the focus groups (eg, adapting the FCF Project to the partner church), and ensured that they reflected participants’ perspectives (step 5). Finally, they met to discuss and finalize the 4 themes (step 6). As part of steps 5 and 6, the 2 researchers used a collaborative and reflexive approach to deepen their interpretations of the data, focusing on achieving “richer interpretations of meaning, rather than attempting to achieve consensus of meaning.”(^62^) To ensure the trustworthiness of the findings, an additional researcher who had helped to facilitate the focus groups reviewed the reviewed the final themes. Credibility was further strengthened through peer debriefing^63^ and by referencing field notes and observations.

### Phase 2

For the quantitative survey data, univariate descriptive analyses of demographic information were first conducted to better understand the church volunteer and caregiver samples. Due to the small sample size, medians were reported instead of means and standard deviations. Nonparametric Wilcoxon signed-rank tests were then conducted to determine whether church volunteers’ knowledge of ADRD changed after the training. These analyses were repeated for caregiver participants to examine whether the main outcomes changed following participation in the FCF intervention. The significance level (α) was set at 0.10. All analyses were conducted using SPSS 29.

### Phase 3

Phase 3 followed the Phase 1 thematic analysis process^60^ (see Phase 1 data analysis).

## Results

### Phase 1

Using thematic analysis,^60^ we identified 4 themes from the focus groups that helped to inform the FCF Project: *emphasizing myths and facts, referrals/resources, communication with healthcare professionals*, and a *dementia friendly church.*

### Emphasizing the Myths and Facts

Across the focus groups, participants shared that the FCF Project should emphasize the myths and facts about AD/ADRD. For example, one participant shared “I think that it [Faith Care Family Project] will be helpful because when my family first started the journey with my mom’s Alzheimer’s. Um. It was like, I mean, we had heard of Alzheimer’s, but you know, you don’t really get into it until it hits home. So. You know, knowing about the diagnosis and the treatment would be beneficial…”

Another participant added, “…making sure that you separate, facts, is really important. I think that’s very important and you much needed especially in the African American church, I know that.”

Additionally, a participant shared, “I’m excited to just get the information and to erase the myths that I probably have already because we hear a lot of things that are not factual.”

### Referrals/Resources

Including referrals and resources to family caregivers was also identified as an important aspect of the FCF Project in the focus groups:

Yes, that would be nice to have our referrals in the manual because some families may not know how to get around and they may not have a doctor or anyone, you know, providing medical assistance for the patient. So I would put it in…let them know that this is where you need to go to get help. Some people, some people just don’t know how to get help and you know that’s OK you if you don’t know you don’t know. So that would be a reference kind of like hey, this is where you go for this or that, something like that.

Similarly, a participant shared:

…it would have been nice to know you know, to have information and resources…because as I'm just speaking for myself, we didn't know, we didn't know what to do [about dementia]. About the range of emotion, the wandering or anything. So just to have [resources] and if we could have got that from the church that would have been helpful too because I'm quite sure a lot of people go through that silently. And no one knows what's going on.

### Communication with Healthcare Professionals

The importance of communication with healthcare professionals was highlighted as need for the FCF Project. For example, one participant shared,

…getting that together as far as dealing with the healthcare professionals because a lot of times you, you'll talk with people and they. You know, they really don't. There's not a great deal of communication that's taking place and it really creates a gap and a void there. And a lot of things kind of fall through the cracks. So I think this [module on communicating with healthcare professionals] is very, very good.

Another participant stated:

…sometimes a doctor may prescribe a medication that he thinks will work for that patient, but it may not. So, we have to help that caregiver to be bold, to speak out, that when he takes this medication, it does ‘X,Y,Z’ things to him. So, I think this is a place where we can really help that caregiver help the patient by speaking out; to not be timid, this is the time to actually speak out…if we need to help them word, whatever they need to say to the doctor or that health care professional, we can do that.

### Dementia Friendly Church

Finally, participants talked about the potential for their church to become “dementia friendly” and a desire to have the FCF Project be part of their efforts to do so:

“There are some people that look to the church for guidance, and I think this is I think this is really practical and really useful and I'm looking forward to it. And now I'm looking forward to that day where we can legitimately say that we're a dementia friendly ministry.”

Similarly, another participant shared, “I’m excited for the journey and the things that I’m learning…so that we can be more dementia friendly as a church, to help families and those who may be going through those things.

Additionally, a participant stated, “it has to start with us, and you know…we would love to eventually be that model church that is dementia friendly.”

The results from the focus groups informed the FCF Project training and implementation, which was adapted from a face-to-face, volunteer-led intervention for African American AD/ADRD family caregivers.^42,43^

### Phase 2

The results of the quantitative data for the church volunteers indicated overall significant improvements in the sum scores of ADKS after the training (*p* = .008) as well as after the intervention (*p* = .05). Some specific ADKS items showed a significant increase after the training including items related to high cholesterol, using reminder notes, and mental exercise as well as after the intervention related to prescription medication (See Table 5).

**Table 5.** Percentage of Respondents to ADKS Scale (N = 9)

	Pretest (pre)	Post training (mid)	Post intervention (post)	Significance *P* values		
ADKS scale	Percentage (Frequency)	Percentage (Frequency)	Percentage (Frequency)	Pre-mid	Mid-post	Pre-post
1. People with AD are particularly prone to depression	77.8 (7)	100.0 (9)	66.7 (6)	0.157	0.083	0.317
2. Mental exercise can prevent a person from getting AD.	55.6 (5)	100.0 (9)	77.8 (7)	0.046	0.157	0.317
3. After symptoms appear, life expectancy is 6-12 years	77.8 (7)	88.9 (8)	77.8 (7)	0.317	0.317	1
4. Health problems can cause agitation	88.9 (8)	88.9 (8)	100.0 (9)	1	0.317	0.317
5. People with AD do best with simple instructions	100.0 (9)	100.0 (9)	100.0 (9)	1	1	1
6. Caregivers should take over right away	22.2 (2)	33.3 (3)	33.3 (3)	0.564	1	0.564
7. Physical activity in the day can reduce daytime behaviors	88.9 (8)	100.0 (9)	88.9 (8)	0.317	0.317	1
8. People have recovered from Alzheimer’s disease	77.8 (7)	100.0 (9)	88.9 (8)	0.157	0.317	0.564
9. Psychotherapy for depression and anxiety can help	100.0 (9)	77.8 (7)	88.9 (8)	0.157	0.564	0.317
10. Sudden trouble with memory and confusion is likely AD.	44.4 (4)	44.4 (4)	55.6 (5)	1	0.564	0.655
11. Most people with AD live in nursing homes	77.8 (7)	88.9 (8)	88.9 (8)	0.317	1	0.317
12. Poor nutrition can make the symptoms of AD worse	77.8 (7)	100.0 (9)	77.8 (7)	0.157	0.157	1
13. People in their 30s can have AD.	88.9 (8)	100.0 (9)	88.9 (8)	0.317	0.317	1
14. As AD progresses people are more likely to fall down	77.8 (7)	100.0 (9)	88.9 (8)	0.157	0.317	0.564
15. It’s helpful to remind them they are repeating themselves	77.8 (7)	77.8 (7)	88.9 (8)	1	0.564	0.564
16. No longer able to make informed decisions about their care	22.2 (2)	33.3 (3)	44.4 (4)	0.317	0.317	0.157
17. Eventually, a person with AD will need 24-hr supervision	100.0 (9)	100.0 (9)	100.0 (9)	1	1	1
18. Having high cholesterol increases risk of developing AD.	55.6 (5)	88.9 (8)	66.7 (6)	0.083	0.317	0.655
19. Tremor or shaking is a common symptom in people with AD.	55.6 (5)	88.9 (8)	66.7 (6)	0.083	0.317	0.705
20. Severe depression can be mistaken for symptoms of AD.	66.7 (6)	100.0 (9)	100.0 (9)	0.083	1	0.083
21. AD is one type of dementia	100.0 (9)	100.0 (9)	100.0 (9)	1	1	1
22. Trouble managing money is an early symptom of AD.	66.7 (6)	88.9 (8)	88.9 (8)	0.157	1	0.317
23. One symptom is belief that people are stealing one’s things	77.8 (7)	100.0 (9)	100.0 (9)	0.157	1	0.157
24. Reminder notes is a crutch that can contribute to decline	66.7 (6)	100.0 (9)	88.9 (8)	0.083	0.317	0.317
25. Prescription drugs that prevent AD are available	55.6 (5)	88.9 (8)	88.9 (8)	0.083	1	0.083
26. High blood pressure may increase risk of developing AD.	55.6 (5)	100.0 (9)	55.6 (5)	0.046	0.046	1
27. Genes can only partially account for the development of AD.	66.7 (6)	77.8 (7)	77.8 (7)	0.564	1	0.317
28. It’s safe to drive with a companion in the car at all times	88.9 (8)	77.8 (7)	100.0 (9)	0.564	0.157	0.317
29. AD cannot be cured	77.8 (7)	100.0 (9)	88.9 (8)	0.157	0.317	0.564
30. Remember recent events better than past events	77.8 (7)	100.0 (9)	77.8 (7)	0.157	0.157	1
Knowledge of ADRD sum score (Mean/SD)	21.67/3.50	26.44/2.01	24.56/3.21	0.008	0.014	0.05

Non-parametric McNemar tests were conducted for each item in the ADKS scale.

Friedman tests were computed to examine sum scores at 3 time points. Post-hoc analyses were conducted to understand differences between timepoints using McNemar tests.

The results of the quantitative data for CG participants showed that caregivers increased their knowledge of ADRD (*z* = −2.68, *p* = .007), improved coping skills (*z* = −2.075, *p* = .038), and increased positive aspects of caregiving (*z* = −1.682, *p* = .092). (See Table 6).

**Table 6.** Key Measurements in Pre- and Post- Tests for Caregiver (N *=* 9)

Key measurements	Pre-test	Post-test	Statistical test, P values*
	Medium (Range)	Medium (Range)	
The AD knowledge scale (ADKS)	22 (14-26)	25 (22-29)	*z* = −2.680, *P* = 0.007
Dementia caregiver burden	52 (23-74)	53 (27-74)	*z* = −0.535, *P* = 0.593
Received social support for caregiving	9 (7-9)	8 (7-10)	*z* = −0.707, *P* = 0.480
Satisfaction with social support	12 (4-16)	15 (4-16)	z = −.0.736, *P* = 0.462
Coping skills	76 (61-88)	83 (63-99)	*z* = −2.075, *P* = 0.038
Positive aspects of caregiving (PAC)	40 (30-45)	43 (34-54)	*z* = −1.682, *P* = 0.092
Cultural justifications for caregiving	32 (29-40)	36 (25-40)	*z* = −0.834, *P* = 0.404
Well-being of caregivers (CWBS)	63 (44-77)	61 (45-76)	*z* = −1.126, *P* = 0.260

*The nonparametric test of Wilcoxon signed-rank tests were conducted.

Following the intervention, all church volunteers and family caregivers were asked to answer several questions evaluating the overall FCF Project and their experience (See Table 7).

**Table 7.** Evaluation of FCF Project

Items	Church volunteers	Caregivers
How would you rate the content of the FCF project?		
Good	0	1 (11.1%)
Very Good	2 (22.2%)	4 (44.4%)
Excellent	7 (77.8%)	4 (44.4%)
How satisfied were you with the FCF project overall?		
Neutral	0	1 (11.1%)
Satisfied	2 (22.2%)	3 (33.3%)
Very Satisfied	7 (77.8%)	5 (55.6%)
I felt confident in making the weekly calls with the caregiver		
Confident	0	N/A
Completely confident	9 (100%)	N/A
The FCF project provided me with relevant information		
Agree	N/A	3 (33.3%)
Strongly agree	N/A	6 (66.7%)
I would recommend the FCF project to others		
Undecided	0	1 (11.1%)
Agree	1 (11.1%)	3 (33.3%)
Strongly agree	8 (88.9%)	5 (55.6%)

### Phase 3

Based on the thematic analysis^60^ described earlier, we identified 4 themes that provide context and additional support for the quantitative findings: *feeling a connection, normalizing the challenges of caregiving, gaining or reinforcing knowledge, and sharing community resources.*

### Feeling a Connection

Participants reported feelings of establishing a connection with one another as part of the study experience. For example, one participant shared:

Yeah, but the strange thing about it, we never met each other, never seen each other. But I sure feel like I know her. Yeah, she says the same about me. Because I told her a lot of stuff that goes on in our family that we never talk about with anybody.” (Diane)

Similarly, a participant stated that although she had never met the volunteer, she felt connected to her:

It's like I just knew her, you know we kind of hit it off and she was very helpful and friendly, you know, discussing the thing, and I would tell her that you know the problem I was having, and she would give me pointers like, you know…She was very helpful.” (Lisa)

Another participant shared that by the end of the 6-week, she felt that her volunteer knew her and her family:

I really did enjoy talking to [volunteer]… I like the fact that she met with me every week and we would discuss various aspects of the chapter. And then she would share with me her experience… then it was like she by the end of the program...it was like she knew me, and she also knew my husband, just from us talking on a weekly basis. (Janey)

### Normalizing the Challenges of Caregiving

A second theme highlighted caregivers expressing that their experiences and feelings were acknowledged and validated by the volunteer, providing reassurance that their challenges were shared by many other AD/ADRD caregivers. One participant indicated:

You know what I learned is that…coping wise…it’s OK to cry…it’s OK to be overwhelmed. And it’s OK to say it. And long as you feel it, see it. Cry it. It’s all OK. And that's the way of coping. (Sheila)

Another participant commented that having a volunteer who had been a caregiver helped them to talk about their experiences without fear of being judged:

I’m also just talking to somebody that’s been there, done that, knows exactly what I’m talking about, you know, has been helpful. I mean, there is no judgment. Or, you know, anything like that. Just honesty, just listening and then offering, you know, some suggestions as to what she did with, you know, her mom and the different stages where she was in. It’s been helpful to me. I've never been through this before. (Pat)

Pat also shared that verbalizing her emotions with a volunteer who had also been an AD/ADRD caregiver made the conversation easier:

And then I tell her, you know, the things that I feel bad about, you know, and I get frustrated and, you know, short tempered on things. You know, just to sit there and talk it through with her and you know her letting me know, you know, that’s kind of like how it was with her, with her mom. But then this is what she did, you know…it’s easier to take something from somebody that's actually been there, done that. (Pat)

### Gaining or Reinforcing Knowledge

A third theme reflected that the participants gained or had reinforced knowledge of AD/ADRD and coping with AD/ADRD caregiving. Some participants reported that the FCF Project volunteer served as a “coach” to help remind them or prepare them for potential behavioral challenges that often come with AD/ADRD caregiving. For example, one participant shared:

…Some things you already experienced and you’re aware of, but it’s like you almost take it for granted…but to be reminded and have that coach remind you, hey, they might do this or that this might happen, or you might if this happens, then you need to react this way just to have that. Reminder that coaching there. (Sheila)

Another participant remarked that they learned “…everyone’s situation is totally different…that it [dementia] comes in different stages.” (Fern)

Other participants indicated the FCF Project reinforced their knowledge of AD/ADRD and that they wished that they had the information earlier in the caregiving role:

And I think it’s a wonderful program. Especially for somebody that’s just starting. It helped me and I've been doing this for like 7 years, but I still learn to things and but for somebody that’s first started now, I think it’s an excellent program. The book is awesome. In my opinion it is. Because I wish I had known all of this stuff when I first started out taking care of my mom. And it's like it just broke it all down. In a nutshell, this is what this is. OK, this is what you expect and expect. You know, it’s in its, you know, it’s not a curable condition, you know…it’s not curable…and it progresses and as it progresses, this is what you can expect. So to me that’s helpful because it doesn't blindside you. (Pat)

Another participant reported that the FCF Project updated their knowledge of AD/ADRD:

Being updated, possibly on some of the things that I may have forgotten…and it really does help to talk to somebody that's experiencing the same thing no matter how much you know, you can always pick up some good solid information. (James)

Some participants shared that they gained knowledge about coping skills including self-care and managing stress. For example, one participant shared:

It [Faith Care Family Project] made me think about taking care of myself a little more, because if I'm not healthy then I'm not healthy enough to take care of my mom. So I know I neglect myself a lot, taking care. I got to take, I have to do better for myself, so that helped me a lot to remind me. (Sheila)

Similarly, a participant shared:

Well, I learned how to cope with my stress better than I used to because I would just be really stressed out and then my head would be hurting and I just kind of feel bad. I kept saying, oh Lord, please don’t let me have a stroke or little heart attack, you know, And I learned how to cope better with that. You know how to relax. (Lisa)

### Sharing Community Resources

The final theme was related to volunteers sharing resources and programs in the community for AD/ADRD caregiver support. For example, one participant shared that her volunteer provided many recommendations for resources:

And so [the volunteer] said…what about [adult] daycares? She said that’s what I did. My mother, she said. You know, just to get her out…and then she gave me a couple of resources, you know, to use to try to find a place, you know, for my mom. And then she told me about a church that does this. It sounds excellent. (Pat)

Another participant reported that learning about resources was new information for her:

That the was very informative and I especially like the listing of different programs that are available because we didn’t even realize that they had places that take people with Alzheimer’s like daycare. We knew nothing about that.” (Kim)

Finally, a participant shared that learning about resources reminder her that she had options for support:

You’re not by yourself and different resources is available in your community to help you and your loved one to you know that just to help the help the family and…and the family members going through dementia. (Beverly)

## Discussion

The primary objective of this study was to pilot test a lay provider, peer-led psychoeducational intervention for African American AD/ADRD caregivers using a multi-phase, mixed methods design. The study comprised 3 phases: (1) focus groups with faith leaders and parishioners to guide the development of a culturally relevant intervention; (2) a face-to-face training with church volunteers and telephone-based intervention with AD/ADRD caregivers; and (3) follow-up interviews with family caregivers to explore their experiences with the intervention.

The Phase 1 findings indicate that the focus groups were an effective approach for engaging church leaders and members, fostering rapport, promoting collaboration, and ensuring culturally appropriate strategies for the FCF Project. In line with their revised sociocultural stress and coping model, Knight and Sayegh^37^ highlight the importance of incorporating group-specific or local cultural values. Focus group participants emphasized the importance of dispelling myths and misconceptions about AD/ADRD. This has also been the emphasized by other church-based studies of AD/ADRD with African American families.^23,29^ Referrals and resources in the community were also highlighted by focus group participants. Previous studies suggest that this is an important topic area to include in AD/ADRD interventions for African American AD/ADRD caregivers as they often underutilized formal services and supports.^64,65^ Moreover, including referrals and resources as part of a faith-placed program may help to overcome barriers related to trusting external or formal sources of support.^34,64-66^

Similarly, communicating with healthcare professionals was mentioned by focus group participants as an important FCF Project module. This is a key finding as African American AD/ADRD caregivers often report experiencing discrimination when seeking healthcare and when managing health care settings for their care recipient with AD/ADRD.^34^ Additionally, the desire to be a dementia friendly church was also shared by focus group participants. These findings support current initiatives to create dementia friendly congregations in the African American community^67^ and the on-going emphasis on African American faith communities to support families affected by AD/ADRD.^23^

The findings from Phase 2 FCF Project church volunteer training and after the intervention showed improvements in participants’ overall knowledge of AD/ADRD. These findings are promising, as there is a continued need for culturally relevant interventions to promote knowledge and dispel myths about AD/ADRD.^68^ Education and training within African American faith communities has also been identified as a platform for addressing misconceptions about AD/ADRD among congregants and the larger community.^29^ The FCF Project training results further corroborate findings from previous studies suggesting that trained volunteers may play a valuable role in delivering interventions for persons living with AD/ADRD and their family caregivers.^69-71^

Both the quantitative findings from Phase 2 intervention and qualitative findings of Phase 3 showed that caregivers increased their knowledge of AD/ADRD. These results are encouraging, given that misconceptions about AD/ADRD, including that cognitive decline and memory loss are a normal part of aging rather than a disease,^2^ can contribute to barriers in seeking care in the African American community. Participants also reported reinforced knowledge of AD/ADRD. These findings highlight the need for timely information about AD/ADRD, even among caregivers who have existing knowledge about the disease. Given that the internet and social media is a frequent source of misinformation about AD/ADRD,^72^ it is important to provide on-going, up to date, and accurate information to family caregivers. Findings also suggested improved caregiver coping skills as part of the Phase 2 intervention. This is notable, as positive coping is fundamental to helping manage AD/ADRD caregiver stres,^73^ including among African American caregivers.^3^ Consistent with previous research, the FCF Project emphasizes that social support and spirituality are culturally relevant, positive coping strategies for African American AD/ADRD caregivers.^74^

Phase 2 results suggested improved positive aspects of caregiving among participants following the FCF Project. Enhancing the positive aspects of caregiving for African American AD/ADRD caregivers through a faith-placed intervention such as the FCF Project may promote resilience and overall caregiver well-being. Research suggests that some African American AD/ADRD caregivers report better psychological well-being compared to other racial groups, which may be linked to their higher levels of religiosity and familism.^75^ The FCF Project also encourages family caregivers to reflect on feelings of personal growth and the dyadic relationship, which may be related to positive aspects of caregiving.^76^

The Phase 3 qualitative findings suggested that the family caregivers felt connected with their trained volunteer. This mirrors other studies showing that trained volunteers can serve as trusted allies who offer empathy, guidance, and practical assistance for AD/ADRD family caregivers.^71,77^ Additionally, findings support previous research indicating that peers or lay providers perceived as cultural ‘insiders’ may establish rapport with participants more easily than professionals.^13,71,78^ Another key finding was that the participants felt that a connection was made with their volunteer despite the “remote” interaction via telephone. These results align with other studies using volunteers in telephone-based programs for older adults,^79,80^ suggesting that the telephone-based aspect of FCF Project was not a barrier to making a positive social connection for the AD/ADRD family caregivers.

Normalizing the challenges of caregiving was a theme in the Phase 3 interviews. Given the reluctance to talk about caregiving ‘stress’ among some African American caregivers,^81^ normalizing and validating feelings of stress may help to encourage open communication. Moreover, given that stigma, cultural concerns, and reliance on family networks are cited as barriers to African American AD/ADRD caregivers seeking formal or external services,^82^ normalizing feelings of caregiver stress may promote receptiveness to utilizing community supports. Similarly, sharing community resources was a theme in Phase 3. Consistent with previous research, learning about community resources from a trusted source may promote using formal resources among African American AD/ADRD caregivers.^66^

Overall, the caregiver–church volunteer relationship that emerged for some participants in the FCF Project represents a distinctive form of dyadic support that may hold particular significance for African American AD/ADRD caregivers. The faith-placed setting of the intervention likely played a central role in shaping this relational dynamic. Historically, predominantly African American churches have served not only as a spiritual anchor but also as a trusted, culturally congruent space for social support, guidance, and health promotion within African American communities.^24,26,29^ Embedding the FCF Project within this setting likely enhanced relational trust, reduced stigma, and fostered a sense of solidarity between caregivers and church volunteers. Furthermore, consistent with the Updated Sociocultural Stress and Coping model,^37^ which emphasizes how cultural identity, values, and informal support systems influence the caregiving experience, this dyadic connection may have supported caregivers’ emotional coping and resilience by normalizing their experiences and reinforcing culturally meaningful forms of support.

### Limitations

Study findings should be interpreted with caution due to several limitations. First, the final sample size of volunteer-family caregiver dyads was very small. This was due in part to the partnership with a smaller African American congregation which posed challenges for caregiver participant recruitment. Partnering with larger churches to implement the FCF Project in the future may help to overcome barriers to recruitment. Second, the sample included African American participants from one metropolitan area in North Texas which limits representation to other geographic areas. Finally, an experimental research design in the future is needed to strengthen the conclusions found in the pilot study of the FCF Project.

## Implications

The findings from the FCF Project offer several contributions to the literature on culturally responsive AD/ADRD caregiving interventions. First, the study reinforces the value of mixed methods research designs^38^ in the development and evaluation of community-based interventions. The use of a three-phase approach enabled both the generation of culturally grounded content and the meaningful exploration of participant experiences. Second, this work adds to the growing evidence base supporting the feasibility and acceptability of faith-placed interventions for African American AD/ADRD caregivers.^24,29^ The integration of trained lay volunteers within the structure of African American churches illustrates a novel dyadic support model that is both culturally congruent and potentially sustainable. Furthermore, the study underscores the importance of leveraging trusted community institutions as sites for intervention delivery and as platforms for future caregiver research.^83^

For clinical practice, the FCF Project offers an innovative model for extending AD/ADRD caregiver support beyond formal healthcare systems. By embedding the intervention within the church, the program addressed known barriers to help-seeking among African American caregivers, including stigma, mistrust, and limited access to culturally appropriate services.^24,84^ The involvement of trained volunteers as supportive partners provided caregivers with a relational source of emotional affirmation, stress normalization, and practical guidance, all of which may complement more traditional medical and psychosocial services. This approach highlights the potential for church-based volunteers to function as culturally attuned, community-embedded extensions of the healthcare team.

At the policy level, the FCF Project points to the continued need for sustained investment in culturally responsive, community-based AD/ADRD care models. Policymakers should consider mechanisms to fund, scale, and evaluate similar lay provider programs, including the development of standardized training, certification, or stipends for volunteers. In addition, the findings support broader calls to integrate faith-based organizations into formal referral networks and long-term care planning efforts.^85^

### Conclusion

The FCF Project pilot findings provide support for future studies with additional African American churches and AD/ADRD family caregivers. Although other studies have examined the use of the African American church as a platform for AD/ADRD family caregiver interventions,^24^ the FCF Project is one of the only telephone-based studies using trained church volunteers as ‘interventionists.’ The results of this study can be used to provide the needed evidence to support the potential call for allocating health care resources effectively to meet the needs of aging adults in community settings. Specifically, investing in faith-based organizations is an innovative way to promote health and build awareness in areas where communities are overburdened.^86,87^ As in the current study, there is also the potential for cost-savings associated with implementing this study design. Finally, integrating the FCF Project into faith communities with a desire to support families caring for loved ones with AD/ADRD may help to broaden its reach to African American AD/ADRD family caregivers in the future.

## Data Availability

The datasets generated during the current study are available from the corresponding author upon reasonable request.*
